# Effects of Drop Sets on Skeletal Muscle Hypertrophy: A Systematic Review and Meta-analysis

**DOI:** 10.1186/s40798-023-00620-5

**Published:** 2023-07-31

**Authors:** Lena Kristiansen Sødal, Eirik Kristiansen, Stian Larsen, Roland van den Tillaar

**Affiliations:** Department of Sport Sciences and Physical Education, Nord University, Levanger, Norway

**Keywords:** Strength training, Resistance, Descending sets, Breakdown sets, Training volume

## Abstract

**Background:**

One of the most popular time-efficient training methods when training for muscle hypertrophy is drop sets, which is performed by taking sets to concentric muscle failure at a given load, then making a drop by reducing the load and immediately taking the next set to concentric or voluntary muscle failure. The purpose of this systematic review and meta-analysis was to compare the effects of drop sets over traditional sets on skeletal muscle hypertrophy.

**Methods:**

This systematic review followed the Preferred Reporting Items for Systematic Reviews and Meta-Analyses guidelines. The SPORTDiscus and MEDLINE/PubMed databases were searched on April 9, 2022, for all studies investigating the effects of the drop set training method on muscle hypertrophy that meets the predefined inclusion criteria. Comprehensive Meta-Analysis Version 3 (Biostat Inc., Englewood Cliffs, NJ, USA) was used to run the statistical analysis. Publication bias was assessed through visual inspection of the funnel plots for asymmetry and statistically by Egger’s regression test with an alpha level of 0.10.

**Results:**

Six studies met the predefined inclusion criteria. The number of participants in the studies was 142 (28 women and 114 men) with an age range of 19.2–27 years. The average sample size was 23.6 ± 10.9 (range 9–41). Five studies were included in the quantitative synthesis. Meta-analysis showed that both the drop set and traditional training groups increased significantly from pre- to post-test regarding muscle hypertrophy (drop set standardized mean difference: 0.555, 95% CI 0.357–0.921, *p* < 0.0001; traditional set standardized mean difference: 0.437, 95% CI 0.266–0.608, *p* < 0.0001). No significant between-group difference was found (standardized mean difference: 0.155, 95% CI − 0.199 to − 0.509, *p* = 0.392).

**Conclusions:**

The results of this systematic review and meta-analysis indicate that drop sets present an efficient strategy for maximizing hypertrophy in those with limited time for training. There was no significant difference in hypertrophy measurements between the drop set and traditional training groups, but some of the drop set modalities took half to one-third of the time compared with traditional training.

## Key Points

• This systematic review highlights the effects of drop sets on skeletal muscle hypertrophy
• In general, there was no significant difference in hypertrophy measurements between the drop set and traditional training groups
• In the studies reporting training duration, drop set modalities took around half to one-third of the time compared with traditional training.

## Background

Resistance training is the primary way of stimulating muscle hypertrophy [1] and, therefore, is an important component in sports training and rehabilitation. Despite the positive outcomes of performing hypertrophy-oriented resistance training, several people refrain from resistance training due to lack of time, which is one of the most frequently self-reported barriers to engaging in physical activity [2] and thereby strength training. Typical traditional hypertrophy-oriented strength training involves resistance exercises of all muscle groups with 2–4 sets of 8–10 exercises with 3–12 repetitions and 2–5 min rest between sets performed 2 and 4 times per week [3]. When including a warm-up and stretching, training traditional strength training often lasts for over an hour in length per training session [3]. Therefore, time-efficient strength training methods have become more popular than ever because they can roughly halve the training time compared with traditional training methods [3].

Researchers have hypothesized that mechanical tension and metabolic stress are mechanisms that increase muscle protein accretion and thereby promote muscle hypertrophy [4]. Conceivably, mechanical tension may be the most important factor and is induced by muscle fiber force generation and stretch [4]. However, metabolic stress, which is an exercise-induced accumulation of metabolites, has also been proposed to play a role in the process of muscle hypertrophy [5]. Several factors have been theorized to play a role in the hypertrophic role of resistance training-induced metabolic stress, such as myokine production [6], cell swelling [4], metabolite accumulation [7], and enhanced fiber recruitment [8].

Furthermore, it has been hypothesized that training for concentric muscular failure may be beneficial when the goal is to maximize muscle hypertrophy [9]. One definition of muscular failure is “the point in a resistance exercise set when the muscle can no longer produce enough force to control the given load” [9]. Theoretically, performing a set to concentric muscular failure may activate all motor units, which may be important because this may have the greatest potential for muscle hypertrophy [10]. Nevertheless, a muscle may not be maximally fatigued when it reaches concentric failure at a given load because force may be produced at lower loads. Therefore, there is a logical rationale for using drop sets from a hypertrophic standpoint because this could fatigue the muscle more than a traditional set which may be beneficial for muscle hypertrophy [11]. There is no clearly defined method of how drop sets are performed in the literature. But the strategy is often used by performing a resistance exercise to concentric muscular failure and immediately performing another set to concentric muscular failure with a load reduction at around 20–25% [10]. The protocol may perform one or several load drops.

Also, increasing the time under load by performing drop sets could promote an additive hypertrophic effect because it may elevate metabolic stress and heighten the sustained compression of vessels. Elevating metabolic stress and heightening the compression of vessels may increase local ischemia and have been proposed as mechanisms for resistance exercise-induced muscle hypertrophy [12]. However, drop set has also been reported to impair neuromuscular performance compared to traditional sets, potentially increasing recovery periods [13], which may result in a decrease in training frequency for the same muscle group.

Although drop sets could be a time-efficient and effective training strategy for enhancing muscle hypertrophy, the strategy has not been well-researched. A small number of studies with inconsistent findings have been carried out to compare the effects of drop sets vs. traditional sets on muscle hypertrophy [14–18]. Therefore, it may be important to synthesize the literature to draw conclusions on the current literature. Thus, the purpose of this systematic review and meta-analysis was to summarize the literature to compare the effects of drop sets over traditional sets on muscle hypertrophy. It was hypothesized that training with drop sets results in similar muscle hypertrophy, but with a shorter resistance training duration compared to traditional training routines.

## Methods

### Literature Search

The SPORTDiscus and MEDLINE/PubMed databases were searched on April 9, 2022, for all studies investigating the effects of drop set training on muscle hypertrophy. The literature search was performed using the following search syntax: “Drop set” OR “Drop set training” OR “Drop set method” OR “Drop-set” OR “Descending sets” OR “Breakdown sets.”

A secondary search was performed by screening reference lists and forwarding searches on Google Scholar. The literature search was exported to Rayyan (https://www.rayyan.ai/), where the initially identified study titles and abstracts were screened for the predefined inclusion criteria. Where a decision based on title/abstract was not possible, a full-text search was performed. This systematic review followed the Preferred Reporting Items for Systematic Reviews and Meta-Analyses (PRISMA) guidelines [19].

### Eligibility Criteria

The review includes studies that: (1) had an experimental design; (2) were published in peer-reviewed, English-language journals; (3) compared drop sets and traditional resistance training regarding estimated changes in muscle mass; (4) had a minimum duration of 6 weeks; (5) only included healthy individuals > 18 years of age; and (6) had a TESTEX score above 6, which is considered “fair quality” or above “fair quality.”

### Data Extraction

The first author’s name and year of publication, participants, sexes, training status, duration of intervention (in weeks), exercise prescription, overview of the training program, measurement variables, and assessment of hypertrophy (pre–post means ± standard deviations) were extracted and tabulated on a predefined Microsoft Excel coding sheet (Version 16.57). During data extraction, it was noticed that two studies [14, 20] used the same data. Therefore, only the data from the 2017 study [14] were included in order to avoid double counting.

### Methodological Quality

A 12-point TESTEX scale was used to assess the methodological quality of the identified studies owing to the specificity of the exercises [21]. The scale contained 12 questions, but a study can receive a total of 15 points due to some questions having several parts. The scale is divided into two categories: The first 5 points given are for study quality, and the last 10 points given for reporting. If the study fulfilled the TESTEX question criteria, they received 1 point; if not, 0 was given. If there was uncertainty about whether the criteria were fulfilled, 0 was given. Studies were classified based on a summary of the scores: “poor quality” (< 6 points), “fair quality” (6–8 points), “good quality” (9–11 points), or “excellent quality” (12–15 points).

### Statistical Analysis

Comprehensive Meta-Analysis Version 3 (Biostat Inc., Englewood Cliffs, NJ, USA) was used to run the statistical analysis. The initial analysis examined whether the drop set and traditional set groups significantly increased their muscle sizes from pre- to post-measure. Then, a between-group comparison of the drop set and traditional set groups was performed. Hedges’ *g* was selected as the measure of effect size to correct for bias regarding small sample sizes [22] and was calculated using pre–post mean changes in muscle size, pooled group standard deviations, number of participants, and the pre–post correlation coefficient. Forest plots were made using random effect modeling to present the test statistic Hedges’ *g* and 95% confidence intervals across the studies, using the inverse variance method to minimize the uncertainty of the pooled effect estimate [23]. Since the correlation coefficient was required to perform the analysis but none of the included studies reported the correlation coefficient or had open datasets, similar studies with open datasets [24, 25] were examined for the correlation coefficients as proposed in Borenstein et al. [22]. As both of these studies showed very high correlations of > 0.88, a more conservative estimate of 0.8 was set across all studies. If the analysis showed significant results (*p* < 0.05), a sensitivity analysis was carried out to determine whether the results were robust, with the lower and even more conservative correlation coefficients of 0.7 and 0.5 [23].

If a study had multiple time points when muscle size was measured, only the pre- and post-measurement values were used for analysis. For those studies assessing muscle hypertrophy in more than one location, measurements were inserted as multiple outcomes and pooled (using the mean of outcomes) into one effect size per study to prevent unit-of-analysis error [23]. The study’s heterogeneity was assessed using Cochran’s *Q* test and *T*^2^ and *I*^2^ statistics with an alpha level of *p* ≤ 0.05 [22]. *I*^2^ values of 0–40%, 40–70%, and > 70% represent low, moderate, and considerable heterogeneity, respectively [23]. Publication bias was assessed through visual inspection of the funnel plots for asymmetry and statistically by Egger´s regression test with an alpha level of 0.10 [22]. If evidence of publication bias was noted, the trim-and-fill method was applied [26]. Sensitivity analysis was also performed, to check if any of the individual studies had a large impact on the results, by removing one study at a time and re-running the analysis. Additionally, since Varović et al. [18] did not equate training volume, we reported the sensitivity analysis from this study in the results since volume is an important driver of muscle hypertrophy [27], which may have confounded the results. Moreover, only the high load group (80%) in Ozaki et al. [17] was included in the quantitative analysis because they reported no differences in training volume between the high load group and the drop set group [17].

The alpha level for the meta-analysis was set to 0.05 to function as a criterion for statistical significance, and trends were declared between 0.05 and < 0.10. Effect sizes of 0–0.2, 0.2–0.5, 0.5–0.8, and > 0.8 were considered to be small, medium, large, and very large, respectively [28].

## Results

### Results of the Literature Search

**Fig. 1:**
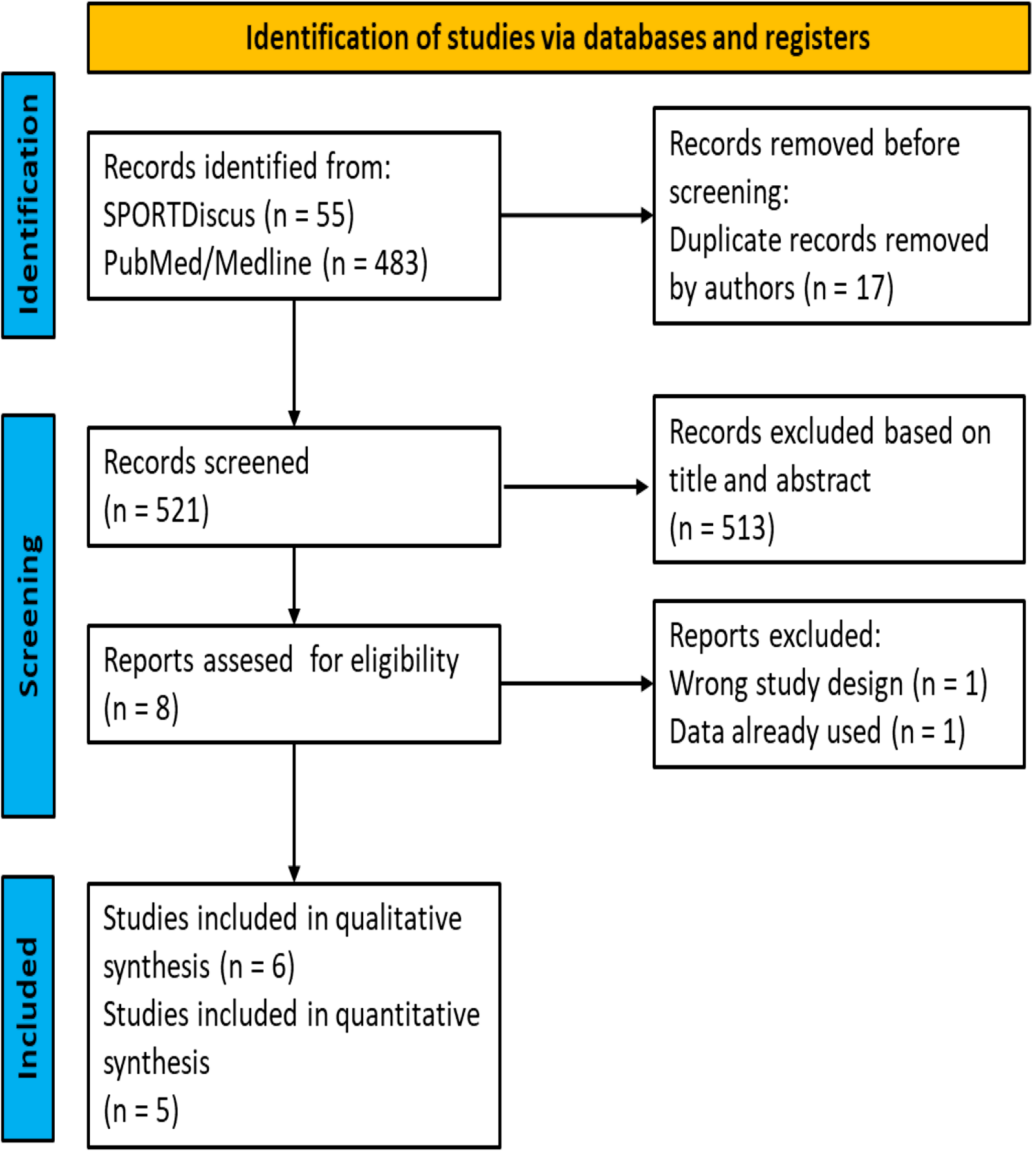
A schematic representation of the search process to find eligible studies for this review. A PRISMA flowchart was used to illustrate the inclusion and exclusion criteria applied in this review

A total of 538 studies were initially identified, which reduced to 521 after duplicates were removed. After the titles and abstracts were screened, 513 studies were excluded for not fulfilling the inclusion criteria. Eight studies were, therefore, assessed for eligibility [14–18, 20, 29, 30]. One study was excluded because of the study design [30], and another was excluded because the data had been used [20]. One study was excluded from the quantitative synthesis due to lack of information [29]. Thereafter, all the reference lists of the included studies were examined for potential studies that might have been missed in the initial search, but no additional studies were found. The search process is summarized in Fig. 1.

### Subjects

The total number of participants in the studies was 142 (28 women and 114 men), with an average sample size of 23.6 ± 10.9 (range 9–41). Five studies included only men [14–18], and one study included both men and women [29]. One study involved untrained participants [17], and the rest of the studies involved active/resistance-trained participants. The duration of the studies ranged from 6 to 12 weeks, with an average of 9 ± 2.23.

The age, body mass, and height ranges were 19.2–27 years, 65.1–81.6 kg, and 171–183.1 cm, respectively.

All six studies assessed muscle hypertrophy: Three measured muscle size by ultrasound [14, 15, 18], two used magnetic resonance imaging (MRI) [16, 17], and one study assessed lean mass using a Bod Pod [29]. Two studies assessed hypertrophy in the arms [16, 17], three assessed hypertrophy in the legs [14, 15, 18], and one study assessed whole body lean mass [29]. The characteristics of the studies are summarized in Table 1.

**Table 1:** Overview of the intervention studies assessing drop sets and the effect upon muscle hypertrophy

Reference	Sample (*N*)	Sex	Training status	Duration (weeks)	Exercise prescription	Training frequency	Condition	Hypertrophy measurement	Main findings
Ozaki et al. [17]	9 (within-subject design)	Men	Untrained	8	Dumbbell curls	2–3 times a week	HL: s, 3; *I*, 80%; *r*, to CF; *p*, 3 min LL: s, 3; *I*, 30%; *r*, to CF; *p*, 90 s DS: s, 1; *I*, 80–65–50–40–30%; *r*, to CF	MRI	Both groups significantly increased CSA, but there was no time × group effect
Fink et al. [16]	16	Men	Active	6	Triceps push-down	Twice a week	Trad: s, 3; *r*, 12 to CF; *I*, 12 RM; *p*, 90 s DS: *s*, 1; *r*, 12; *I*, 12 RM to CF, 3 drops at 20% each	MRI	Both groups significantly increased CSA, but there was no time × group effect
Fisher et al. [29]	41	Both	Resistance trained	12	Chest press, leg press, pull-down	Twice a week	Trad: s, 1; *r*, 8–12 to CF DS: *s*, 1; *r*, 8–12 to CF, 1 drop at 30% HLDS: *s*, 1; *r*, 4 to CF, 2 drops at 20% each	Bod Pod	No significant changes in body composition were found in either of the groups
Angleri et al. [14]	32 (within-subject design)	Men	Resistance trained	12	Leg press, leg extension	Twice a week	Trad: *s*, 3–5; *r*, 6–12 to CF; I, 75%; *p*, 2 min CP: s, 3–5; *r*, 6–15 to CF; *I*, 65–85%; *p*, 2 min DS: *s*, 3–5; *r*, to CF; *I*, 50–75%, 1–2 drops at 20%; *p*, 2 min	Ultrasound	Both groups significantly increased CSA, but there was no time × group effect
Varović et al. [18]	16 (within-subject design)	Men	Recreationally active	8	Unilateral leg extension	1–3 times a week	Trad: *r*, to CF; *I*, 15 RM; *p*, 120 s DS: *r*, to CF; *I*, 5 RM, 1 dro*p* at 20%, 1 drop at 10–15%	Ultrasound	Both Trad and DS significantly increased muscle thickness in VL and RF. In RF at 30% and 50% muscle length, there was also a time × group effect favoring DS
Enes et al. [15]	28	Men	Resistance trained	8	Back squat, leg press, leg extension	Twice a week	DS: *s*, 3; *r*, 10 + 6; *I*, 75 + 55%; *r*, 120 intersets RP: *s*, 3; *r*, 10 + 6; *I*, 75%; *p*, 20 s intrasets, 120 s interset Trad: *s*, 4; *r*, 12; *I*, 70%; *p*, 120 s interset	Ultrasound	Both Trad and DS significantly increased muscle thickness in the proximal and middle VL, but not in the distal part. There was no time × group effect

*DS* drop set; *Trad* traditional set; *HL* high load; *LL* low load; *RP* rest pause; *CP* crescent pyramid; *CSA* cross-sectional area; *s* set; *r* repetition; *p* pause; *I* intensity (percentage of 1 repetition maximum); *CF* concentric failure; *RM* repetition maximum; *intraset* between repetitions; *interest* between sets; *MRI* magnetic resonance imaging; *VL* vastus lateralis; and *RF* rectus femoris

### Quality Assessment

The results from the TESTEX quality assessment are presented in Table 2. The scores ranged from 7 to 10, with an average score of 8.5 ± 1.3. Three studies were rated “fair” quality [14, 16, 17], and the other three were rated “good” quality [15, 18, 29]. None of the studies were rated “poor” or “excellent” quality based on the TESTEX criteria.

**Table 2:** TESTEX quality assessment

	1	2	3	4	5	6a	6b	6c	7	8a	8b	9	10	11	12	Total score (max. 15)
Ozaki et al. [17]	0	0	1	1	0	1	0	1	0	1	1	0	0	1	1	8
Fink et al. [16]	0	0	1	1	0	0	0	0	0	1	1	1	0	1	1	7
Fisher et al. [29]	1	1	1	1	0	0	1	0	0	1	1	1	0	1	1	10
Angleri et al. [14]	1	0	1	1	0	0	0	0	0	1	1	0	0	1	1	7
Varović et al. [18]	1	0	1	1	1	0	1	0	0	1	1	1	0	1	1	10
Enes et al. [15]	1	0	1	1	0	1	0	0	0	1	1	1	0	1	1	9
Sum	4	1	6	6	1	2	2	1	0	6	6	4	0	6	6	

### Pre–Post Within-Group Changes in Muscle Size

**Fig. 2:**
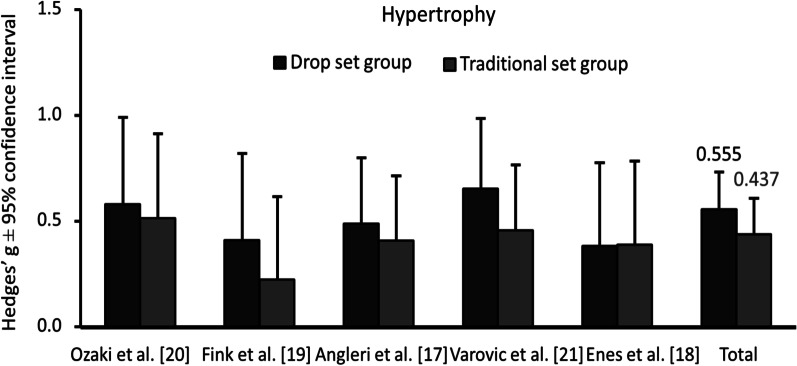
Pre–post changes in muscle size

Both the drop set and traditional set groups increased muscle size from pre- to post-measure (both *p* < 0.0001). The standardized mean difference for the drop set group was 0.555 (95% CI 0.357–0.921), and for the traditional set group, it was 0.437 (95% CI 0.266–0.608), as shown in Fig. 2.

### Between-Group Comparison of Changes in Muscle Size

**Fig. 3:**
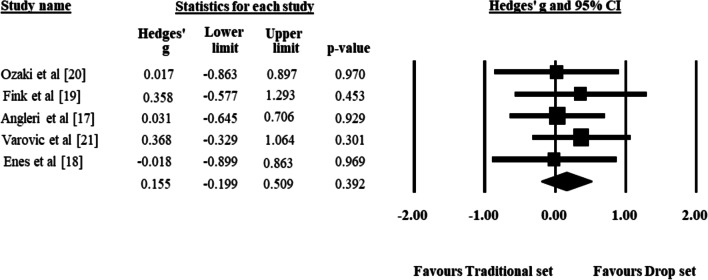
Forest plot of between-group comparison of changes in muscle hypertrophy

The meta-analysis revealed no significant differences in hypertrophy between the drop set and traditional set groups (Fig. 3), with a mean effect size of 0.155 (95% CI − 0.199 to − 0.509; Z = 0.857; *p* = 0.392). One study removal sensitivity analysis showed that none of the individual studies had a large impact on the results. Specifically, when removing Varović et al. [18], which did not equate training volume, we found a mean effect size of 0.08 (95% CI − 0.331–0.492; Z = 0.384; *p* = 0.701).

### Heterogeneity and Risk of Bias

Cochran’s *Q* test for heterogeneity found no significant study variance between the drop set and traditional set groups (*Q* = 0.87, *T*^2^ = 0.00, *I*^2^ = 0.00,* p* = 0.929). Furthermore, Egger´s test for funnel plot asymmetry did not indicate any potential publication bias (*p* = 0.383). Trim-and-fill analysis was, therefore, not carried out.

## Discussion

The purpose of this systematic review and meta-analysis was to summarize the literature to compare the effects of drop sets over traditional sets on skeletal muscle hypertrophy. The main findings were that both the drop set and traditional set training lead to significant pre–post increases in muscle size, but the meta-analysis found no significant differences between groups. The mean length of the included studies was 9 ± 2.2 weeks (range 6–12), which should be sufficient to detect significant increases in muscle size [31].

Drop sets are often used to enhance muscle hypertrophy because decreasing the load may be an effective strategy to fully fatigue the muscle that may enhance muscular adaptations [10]. Additionally, this strategy could increase the time under load, elevating metabolic stress and ischemia, which are proposed mechanisms of muscle hypertrophy [12]. Despite this, our findings showed similar increases in muscle hypertrophy between drop sets and traditional sets. However, two of the five studies included in the quantitative analysis reported training duration time. Both reported significantly shorter training duration (drop set: 145.4 ± 21 s, traditional set: 315.8 ± 42.2 s [16], drop set: 2.1 ± 0.1 min, traditional set: 6.8 ± 0.13 min [17]). Together, this shows that drop-set training may be as effective as traditional sets for increasing muscle size but that it is more time-efficient.

Moreover, Ozaki et al. [17] compared high loads with low loads in a traditional set structure and found no significant differences, but the low load group had to perform over 100 repetitions to reach failure. This resulted in almost double the training time compared to the high load group and the drop set group. Therefore, the high load group acted as a control group in this systematic review and meta-analysis because it is more ecologically valid. Moreover, Fink et al. [16] compared a single drop set with three traditional sets. Interestingly, only the drop set group significantly increased CSA (drop set: 10.0 ± 3.7%, traditional set: 5.1 ± 2.1%) from pre- to post-test. However, they reported no significant between-group differences [16]. It is speculated that the small sample size and short duration (6 weeks) of the intervention could be two reasons why the results were not significant. 

In five of the six studies [14, 16–18, 29], the participants continued to concentric muscle failure, whereas Enes, Alves [15] stated that participants performed the sets to or close to muscle failure. Interestingly, this was the only study where the effect size for the drop set group was not larger than the effect size for the traditional training group [13]. Therefore, it is speculated that performing the drop sets to concentric muscle failure is required for this strategy to be as effective as traditional sets for enhancing muscle hypertrophy.

Despite meta-analysis results failing to show significant differences between groups regarding muscle hypertrophy, one of the studies showed that regional hypertrophy in different parts of the same muscles varied across the modalities. Varović et al. [18] examined hypertrophy in three different parts of the vastus lateralis and rectus femoris using a within-subject design. Both modalities significantly increased the muscle thickness of both muscles. However, there was also a time × group effect at the proximal and middle parts of the rectus femoris, favoring the drop set. These findings correspond to earlier studies, which also show that quadriceps may differ in regional muscle adaptations as a response to exercise [32–34]. This indicates that drop sets could be a more effective strategy for more complete muscle growth of the rectus femoris. Future studies aiming to compare drop sets with traditional sets should, therefore, measure regional hypertrophy in different parts of the muscles.

Angleri et al. [14] randomized 16 men to a drop set or traditional set group, using a within-subject design, to compare the changes in muscle hypertrophy in the vastus lateralis (measured at approximately 50% femur length) using ultrasound, and when the total training volumes for the drop set and traditional set groups were equal. Both conditions significantly increased their CSA but there was no time × group effect. Interestingly, Angleri et al. [20] reported individual increases in CSA from pre- to post-test and reported low subject variability between the drop set and traditional groups (range: 1.7–13.3%) compared to other studies (range: 11–30%) [35, 36]. The authors speculated that this was due to: (1) individuals had an initial training load based on their training history; (2) the 7% increase in training volume every sixth training session, (3) 30 g of whey protein was digested after each workout, and (4) the use of a within-subject experimental design allowing for a precise volume equalization and a minimized effect of between-subject biological variability. Therefore, we postulate that drop sets and traditional sets may result in low inter-individual response if these aspects of the training programs are provided.

### Limitations

One limitation of this systematic review and meta-analysis is that only a few studies have investigated the effects of the drop set method on skeletal muscle hypertrophy. Therefore the results are preliminary. Therefore, it can be difficult to answer the research questions with certainty and generalize to the real-world population in terms of the use of drop sets for skeletal muscle hypertrophy purposes. Due to the lack of studies examining untrained participants, a sub-analysis for trained versus untrained participants was not performed. This is a limitation because untrained participants respond differently due to neurological adaptations. Sub-analysis was also not carried out on different muscles, which could be interesting to investigate if drop sets were to lead to better skeletal muscle growth in the upper versus the lower body. Furthermore, the use of different methods to measure muscle hypertrophy in the included studies could be another limitation which impacts our understanding of the relative benefits of these two resistance training approaches.

#### Practical Applications

Based on the results of this meta-analysis, the choice of whether to incorporate drop sets in the training routine is entirely up to the individual, with personal preferences and limited time for training being primary factors in this choice, because both modalities increased muscle size equally.

Drop sets can be a time-efficient strategy because one can add additional volume without increasing training duration. One can simply add a drop set into the traditional workout to gain a higher volume while keeping the workout short. Since there is a dose–response relationship between volume and hypertrophic responses [37], drop sets can be used to maximize muscle hypertrophy for those with limited time for training.

The choice between drop sets and traditional sets is not a question of either/or. The incorporation of both could potentially yield the best results because some regional parts of the rectus femoris responded significantly better to drop sets than to traditional sets, which indicates that regional hypertrophy in some muscles may favor the stimuli from drop sets, whereas others favor the stimuli from traditional sets.

It would be difficult to formulate specific recommendations on how drop sets should be performed based on the results of this review. However, speculation suggests that repetitions should be performed to concentric muscle failure to ensure maximal effects. Therefore, drop set modalities could be well-suited for machine-based training because of the higher degree of stability [38] and, therefore, the potential lower risk of injury when going to concentric muscle failure.

#### Future Studies

Drop sets have been hypothesized to be a better strategy for stimulating type 1 muscle fibers to muscle growth because of the higher fatigue threshold [1, 10]. As women have a higher proportion of type 1 muscle fibers compared to men [39], it is speculated that women could respond better to drop set modalities because of a higher resistance to fatigue. Future studies should, therefore, address the hypertrophic effect of drop sets in women when confounding variables are accounted for and with regional hypertrophy measurement such as ultrasound, MRI or DXA. Furthermore, different modalities of drop sets should be compared to analyze if multiple drops are more effective than fewer or to determine what intensities should be used for maximal hypertrophic effect.

## Conclusion

The results of this systematic review and meta-analysis indicate that drop sets present an efficient strategy for maximizing skeletal muscle hypertrophy in those with limited time for training. There was no significant difference in hypertrophy measurements between the drop set and traditional set groups, but some of the drop set modalities took half to one-third of the time compared with the traditional set training. Small sample sizes led to low statistical power in some of the studies, which might have been one of the reasons behind the non-significant results because four out of five studies had higher effect sizes, favoring drop sets.

## Data Availability

Please contact the corresponding author for data requests.

## Abbreviations

DS: Drop set
Trad: Traditional set
HL: High load
LL: Low load
RP: Rest pause
CP: Crescent pyramid
CSA: Cross-sectional area
*s*: Set
*r*: Rep
*p*: Pause
*I*: Intensity (percentage of 1 repetition maximum)
CF: Concentric failure
RM: Repetition maximum
intraset: Between repetitions
interset: Between sets
MRI: Magnetic resonance imaging
VL: Vastus lateralis
RF: Rectus femoris
